# Methylenetetrahydrofolate Reductase C677T (rs1801133) Polymorphism Is Associated with Bladder Cancer in Asian Population: Epigenetic Meta-Analysis as Precision Medicine Approach

**DOI:** 10.3390/cancers15174402

**Published:** 2023-09-02

**Authors:** Athaya Febriantyo Purnomo, Besut Daryanto, Kurnia Penta Seputra, Taufiq Nur Budaya, Nurul Cholifah Lutfiana, Fahrul Nurkolis, Sanghyun Chung, Jin Young Suh, Moon Nyeo Park, Byung-Kwan Seo, Bonglee Kim

**Affiliations:** 1 Department of Oncology, University of Oxford, Oxford OX3 7DQ, UK; 2 Department of Urology, Faculty of Medicine, Universitas Brawijaya, Malang 65142, Indonesia; 3 Department of Biosciences and Biomedicine, Faculty of Medicine, Universitas Muhammadiyah Surabaya, Surabaya 36201, Indonesia; 4 Department of Biological Sciences, State Islamic University of Sunan Kalijaga (UIN Sunan Kalijaga), Yogyakarta 55281, Indonesia; fahrul.nurkolis.mail@gmail.com; 5 Department of Pathology, College of Korean Medicine, Kyung Hee University, Hoegidong Dongdaemun-gu, Seoul 02447, Republic of Korea; 6 Kyung Hee Myungbo Clinic of Korean Medicine, Hwaseong-si 18466, Republic of Korea; 7 Seoul Forest Korean Medicine Clinic, Ttukseomro 312, Seongdong-gu, Seoul 04773, Republic of Korea; 8 Korean Medicine-Based Drug Repositioning Cancer Research Center, College of Korean Medicine, Kyung Hee University, Seoul 02447, Republic of Korea; 9 Department of Acupuncture and Moxibustion Medicine, Kyung Hee University College of Korean Medicine, Kyung Hee University Hospital at Gangdong, Seoul 05278, Republic of Korea

**Keywords:** bladder cancer, epigenetic, meta-analysis, methylenetetrahydrofolate reductase, precision medicine

## Abstract

**Simple Summary:**

This research aims to understand how gene variations, particularly in the methylenetetrahydrofolate reductase gene (MTHFR), influence bladder cancer development. The study conducted a meta-analysis of seven relevant studies to assess the impact of a specific genetic variant, rs1801133, on bladder cancer susceptibility. The findings indicate a significant association between the MTHFR rs1801133 polymorphism and bladder cancer risk, especially in the Asian population. People with the T-allele or TT genotype had a higher likelihood of developing bladder cancer compared to that of those with the C-allele. This research is vital for the scientific community as it enhances our understanding of genetic factors contributing to bladder cancer. The findings may lead to targeted prevention and personalized treatment strategies. Additionally, the systematic review and meta-analysis provide a comprehensive overview of the existing evidence, adding to the knowledge base on bladder cancer genetics. This could inspire further research into the genetic basis of complex diseases like bladder cancer.

**Abstract:**

The etiology of bladder cancer remains unclear. This study investigates the impact of gene polymorphisms, particularly methylenetetrahydrofolate reductase gene (MTHFR), on bladder cancer susceptibility, focusing on the rs1801133 single-nucleotide polymorphism (SNP). A meta-analysis was conducted after systematically reviewing the MTHFR gene literature, adhering to PRISMA guidelines and registering in PROSPERO (CRD42023423064). Seven studies were included, showing a significant association between the MTHFR C677T (rs1801133) polymorphism and bladder cancer susceptibility. Individuals with the T-allele or TT genotype had a higher likelihood of bladder cancer. In the Asian population, the overall analysis revealed an odds ratio (OR) of 1.15 (95% CI 1.03–1.30; *p*-value = 0.03) for T-allele versus C-allele and an OR of 1.34 (95% CI 1.04–1.72; *p*-value = 0.02) for TT genotype versus TC+CC genotype. The CC genotype, however, showed no significant association with bladder cancer. Notably, epigenetic findings displayed low sensitivity but high specificity, indicating reliable identified associations while potentially overlooking some epigenetic factors related to bladder cancer. In conclusion, the MTHFR T-allele and TT genotype were associated with increased bladder cancer risk in the Asian population. These insights into genetic factors influencing bladder cancer susceptibility could inform targeted prevention and treatment strategies. Further research is warranted to validate and expand these findings.

## 1. Introduction

Bladder cancer (BC) stands as a significant global health challenge, ranking as the 10th most frequently diagnosed cancer on a worldwide scale. Data from GLOBOCAN’s comprehensive reports for the year 2020 indicate a concerning estimation of 573,000 new cases of bladder cancer, accompanied by a distressing 213,000 fatalities attributed to this disease [1]. Intriguingly, the propensity for men to be afflicted by bladder cancer surpasses that of women. This discrepancy is highlighted by the marked global incidences and mortality rates for men, towering at 9.5 and 3.3 per 100,000, respectively—a staggering four times higher than the corresponding figures for women [1].

Delving into the enigma of bladder cancer’s origins reveals an intricate tapestry of contributing factors. Environmental elements, including smoking and occupational exposure to specific chemicals, have been linked to the disease. The intriguing observation arises within populations exposed to identical environmental conditions—a fraction of individuals, rather than the entirety, appear vulnerable to bladder cancer. This phenomenon tantalizingly hints at a multifaceted etiology in which genetic and epigenetic elements are pivotal players in driving the development of this complex disease [2,3].

Steering the discourse toward the realm of epigenetics opens a gateway to a deeper understanding of the intricate molecular underpinnings of bladder cancer. The term “epigenetics” encapsulates a realm of heritable alterations in gene expression that do not arise from changes in the fundamental DNA sequence. Among these mechanisms, DNA methylation occupies a central role. Esteemed for its status as one of the earliest forms of epigenetic regulation, DNA methylation has garnered attention due to its involvement in developmental processes, aging, and carcinogenesis. This regulatory phenomenon manifests through the hypermethylation of tumor suppressor genes and the hypomethylation of oncogenes, contributing to the complex mosaic of cancer pathogenesis [4,5].

A captivating player within the realm of epigenetic influence is the methylenetetrahydrofolate reductase (MTHFR) gene. Nestled within the one-carbon metabolic pathway (OCM), this gene orchestrates the liberation of unbound methyl groups (CH3), concurrently undertaking the irreversible conversion of 5,10-methylenetetrahydrofolate to 5-methyltetrahydrofolate—a pivotal role in the intricate orchestration of cellular processes [6]. DNA methylation, a crucial facet of epigenetics, is grounded in the addition of a methyl group (CH3) to the 5-carbon of cytosine. This biochemical ballet is choreographed by the concerted efforts of DNA methyltransferase (DNMT) enzymes, culminating in the formation of 5-methylcytosine (5-mC) [6].

Comprehending the pathways of DNA methylation unveils a dual journey of establishment and maintenance. Throughout embryonic development, DNMT3A and DNMT3B commandeer the reins of DNA methylation, contributing to the establishment of intricate patterns. During the replication of DNA, DNMT1 assumes a central role, orchestrating the perpetuation of these patterns across successive cell generations [7]. This process, akin to a cellular memory mechanism, intricately weaves the programming of gene expression into the fabric of cellular identity [8].

Diving deeper into the intricate dance of epigenetics, Ren et al. (2018) present a noteworthy association between the gene polymorphism rs1801133 of methylenetetrahydrofolate reductase and global DNA hypomethylation. This intriguing connection sets the stage for potential quantitative changes at the genetic level, subsequently influencing the likelihood of cancer development [9]. At the heart of this association lies the intricate interplay of DNA methylation and its far-reaching impact on gene expression, ultimately reverberating through the tapestry of health outcomes [9].

DNA methylation is a crucial epigenetic mechanism that involves the addition of a methyl group to specific regions of DNA molecules. It plays a fundamental role in regulating gene expression patterns, essentially determining which genes are turned on or off in a cell. In the given context, two main pathways for DNA methylation are described: the establishment pathway and the maintenance pathway. DNMT3A, DNMT3B, and DNMT1 are enzymes responsible for facilitating DNA methylation during embryonic development and DNA replication. These enzymes are integral to establishing and inheriting DNA methylation patterns across subsequent cell generations [9]. It highlights how the gene polymorphism rs1801133 of MTHFR, leading to global DNA hypomethylation, can result in significant disruptions in gene expression regulation, potentially increasing the risk of developing cancer. This is due to the crucial role that DNA methylation plays in maintaining proper gene expression patterns, and its disturbance can have far-reaching consequences for cellular function and health outcomes [9].

Furthermore, there have been reported potential correlations between single-nucleotide polymorphisms (SNPs) of DNA methyltransferases (DNMTs) and the susceptibility to various types of malignancies. For instance, DNMT3A has been linked to gastric cancer [10], DNMT3B to breast cancer [11] and lung cancer [12]. Based on the aforementioned discoveries, our objective was to assess the influence of rs1801133 SNP located in the methylenetetrahydrofolate reductase genes on the vulnerability to bladder cancer in an Asian population dataset, utilizing both qualitative and quantitative methods. The journey into the realm of bladder cancer uncovers a complex interplay of factors. From its rank as the tenth most commonly diagnosed cancer to its association with genetic and epigenetic contributors, bladder cancer emerges as a multidimensional puzzle. Epigenetics, with its DNA methylation and regulatory mechanisms, adds a layer of depth to our understanding of this intricate disease. The study of gene polymorphisms, such as the MTHFR gene’s rs1801133 variant, illuminates potential avenues for precision medicine. As research continues to unfold, unraveling the complex web of interactions, the horizon of possibilities for targeted interventions and personalized treatments beckons, potentially revolutionizing the landscape of bladder cancer management.

This comprehensive exploration underscores the multidisciplinary nature of bladder cancer research, encompassing epidemiology, genetics, epigenetics, and molecular biology, as we endeavor to decipher the complexity of its etiology and devise novel strategies to combat its impact on global health.

## 2. Materials and Methods

### 2.1. Data Extraction—Inclusion and Exclusion Criteria

The methodology employed in this analysis rigorously adhered to a set of predefined inclusion and exclusion criteria, ensuring the reliability and robustness of the study’s findings. The inclusion criteria encompassed studies that delved into the intricate correlation between methylenetetrahydrofolate reductase SNPs and the susceptibility to bladder cancer development in humans. Specifically, the studies had to adopt a case-control design, providing a systematic framework for examination. Furthermore, these studies were required to furnish comprehensive genotypic frequencies, a pivotal aspect in deciphering the genetic landscape under scrutiny.

Conversely, the study’s exclusion criteria were meticulously established to uphold the integrity of the research. Rigorous scrutiny was applied to filter out any research papers or studies that presented overlapping or duplicate data, thereby safeguarding against redundancy. Similarly, texts that exhibited incompleteness were judiciously excluded to ensure the availability of robust and complete information. Studies that deviated from the categorization as case or control studies were omitted to maintain the alignment of study design. Furthermore, the selection process prioritized studies exclusively linked to bladder cancer, eschewing studies that deviated from the study’s core focus. Importantly, reviews or meta-analyses were purposefully excluded to retain a primary and unbiased source of data.

In the realm of data analysis, it is noteworthy that the precise data and the specific single nucleotide polymorphisms (SNPs) under investigation are currently pending release, emphasizing the meticulous planning required for subsequent stages of analysis.

The implementation of the Hardy–Weinberg equilibrium (HWE) formula was executed with meticulous attention to detail, mirroring the methodology outlined by Rodriguez et al. in 2009 [13]. A rigorous criterion for deviation from HWE was set, requiring a calculated X2 value exceeding 3.84. To ensure the highest level of accuracy, three independent reviewers (AFP, NCL, and BD) collectively embarked on an exhaustive screening process for each record and report. Collaborative discussions among these reviewers further ensured the attainment of consensus and the refinement of the study’s framework. Any discrepancies that emerged during the incorporation of studies were conscientiously resolved through the consensus-building process, upholding the study’s integrity and minimizing bias.

The meticulous design and implementation of these methodologies not only validate the research’s reliability but also underscore the dedication to producing findings that are not only scientifically sound but also methodologically comprehensive.

### 2.2. Strategy for Data Synthesis 

A systematic review of the literature was conducted using the PubMed, Cochrane, Embase, and Google Scholar databases to identify pertinent studies published up until March 2023. The systematic review protocol was written following the Preferred Reporting Items for Systematic Reviews and Meta-Analyses and was also reviewed by the boards of PROSPERO-NIHR (International Prospective Register of Systematic Reviews—National Institute for Health Research) with the number CRD42023423064. The search strategy utilized for the methylenetetrahydrofolate reductase gene involved the use of various combinations of keywords. These include: ((Bladder) AND (cancer)) AND (methylenetetrahydrofolate reductase), ((urothelial carcinoma) OR (bladder cancer)) AND (methylenetetrahydrofolate reductase), (urothelial carcinoma) AND (methylenetetrahydrofolate reductase), (transitional cell carcinoma) AND (methylenetetrahydrofolate reductase gene), (urothelial carcinoma) AND (methylenetetrahydrofolate reductase polymorphism), (methylenetetrahydrofolate reductase polymorphism) AND (transitional cell carcinoma), (bladder cancer) AND (methylenetetrahydrofolate reductase polymorphism), (transitional cell carcinoma) AND (methylenetetrahydrofolate reductase polymorphism) AND (single nucleotide polymorphism), (epigenetic), (mutation gene). The language of communication utilized in the workplace was restricted to English. Additional reports were identified through the references cited within the chosen papers.

The comprehensive examination of records was executed with the utmost rigor, involving the active engagement of all authors working independently. Within this meticulous data extraction process, every relevant piece of information was diligently identified, encompassing critical details such as the first author’s identity, the publication year, the composition of the study population, the nuanced genotyping methodologies employed, the specific genes and SNPs under scrutiny, the discernment of case and control numbers, and the meticulous assessment of adherence to the Hardy–Weinberg equilibrium (HWE). This intricate process, akin to carefully threading together pieces of an intricate mosaic, was meticulously undertaken by a singular author, systematically inputting the gleaned information into Microsoft Excel v2013 (Washington, DC, USA). In instances where the terrain of uncertainty emerged, a proactive approach was adopted by seeking consultation with the other authors, thereby fostering an environment of collaborative synergy. Through a series of iterative deliberations, the nexus of consensus was steadfastly reached, effectively addressing and refining each ambiguous datum. 

Navigating research integrity, proactive efforts were undertaken to establish a direct channel of communication with the primary researchers responsible for the studies. The intention behind this endeavor was twofold: to glean supplementary details that would enrich the data landscape and to acquire complete manuscripts that would serve as invaluable assets in the subsequent stages of analysis. A shared commitment among all authors echoes in the composition of the manuscript, reflecting the collective dedication and intellectual prowess that was harnessed throughout the research journey. It is imperative to underline that the task of analyzing bladder cancer’s intricate epigenetic SNPs panorama will be conducted through the adept utilization of Review Manager 5.4 software (London, UK), a tool housed within the esteemed domain of the Cochrane Library, UK. 

### 2.3. Risk of Bias (Quality) Assessment

The quantitative analytical phase of the study encompassed a rigorous evaluation of each included research article, underpinned by a comprehensive scoring mechanism aligned with the quality assessment criteria as formulated by Li et al. This systematic approach led to the assignment of specific quality scores spanning a spectrum from 0 to 15, thereby providing a nuanced gauge of the methodological and empirical robustness of each study. It is noteworthy that the existing scholarly discourse has indicated that research endeavors achieving a commendable score of 9 on this scale are often attributed with a higher echelon of methodological and empirical quality. Conversely, studies that do not meet this threshold are relegated to the category of exhibiting relatively lower quality standards [14].

Within the analytical methodologies, the study engaged two prominent strategies that play pivotal roles in the analysis. These are the inverse variance method and the DerSimonian–Laird method, each resonating with distinct analytical paradigms. The former method aligns itself harmoniously with the fixed-effect analysis model, catering to situations where homogeneity is a prevailing assumption. In contrast, the latter method, the DerSimonian–Laird method, adheres to the random-effect analysis model, accommodating scenarios where heterogeneity among study effects is a pivotal consideration.

A heightened level of analytical rigor was upheld through a significance level set at 0.05. This threshold, embedded within the analytical framework, assumes paramount significance as it serves as a definitive criterion for the statistical interpretation of results. In consonance with established academic conventions, *p*-values derived from statistical tests that fall below this designated threshold are indicative of statistically significant findings. This practice underscores the meticulousness with which the study’s findings were scrutinized, as only effects attaining this level of significance were deemed substantial within the analytical narrative. The calculations were executed utilizing MetaGenyo [15], which can be accessed at https://metagenyo.genyo.es/ (accessed on 1 June 2023).

### 2.4. Population

The quantitative analysis phase of this research encompassed a comprehensive examination of published data emanating from diverse geographic locales within the ambit of the study’s interest. Within this methodological construct, the study seeks to embark on a rigorous assessment of epigenetic polymorphisms that transpired within the intricate fabric of a multinational study conducted within the Asian continent. This particular focus underscores the research’s commitment to unraveling the nuanced interplay of genetic variations within a broad and multifaceted context, spanning across diverse regions and populations.

### 2.5. Analysis of Subgroups or Subsets

The available dataset from the included studies was computed using Review Manager version 5, which is a software application developed by the Cochrane Library in the United Kingdom. The dataset incorporated within the software was initially gathered using Microsoft Excel v2013. The utilization of a random effect model was implemented upon obtaining a *p*-value of 0.05 in the heterogeneity test. The study utilized the odds ratio (OR) to report findings pertaining to dichotomous data. Additionally, a 95% confidence interval (CI) was calculated. In order for the overall analysis to be deemed statistically significant, a *p*-value of 0.05 was required for interpretation. The I^2^ statistic was employed to assess heterogeneity among the studies that were incorporated.

### 2.6. Condition or Domain Being Studied

The central axis of inquiry for this research was dedicated to the exploration of epigenetic modifications that have unfolded within the Asian population’s genetic terrain, particularly within the sphere of bladder-cancer-associated mutation genes. In this specific context, the spotlight is cast upon the methylenetetrahydrofolate reductase C677T polymorphism (rs1801133), a pivotal genetic locus that has captured the attention of researchers. The overarching objective encompasses the meticulous aggregation of epigenetic revelations stemming from a compendium of previously published scholarly works. This amalgamation is poised to culminate in an intricate pooled analysis, elegantly portrayed through the dynamic medium of forest plots. These visual depictions hold the potential to unveil the gene’s susceptibility dynamics, offering a panoramic insight into its intricate involvement within the tapestry of bladder cancer.

This study took a robust approach to uphold the importance of its findings. Measures were in place to monitor and mitigate any potential sources of bias. The assessment of research quality assumed a pivotal role in this regard, involving rigorous evaluations that scrutinized the empirical robustness and methodological soundness of the incorporated studies. This vigilance extends to the construction of funnel plots that serve as an invaluable visual tool to assess publication bias.

Moreover, the analytical journey was fortified with the deployment of quantitative indices such as I^2^, Egger’s test, and Chi^2^ values. These metrics, generated through utilization of software manager tools, lent empirical weight to the research’s conclusions. Thus, the research unfolds as a meticulously orchestrated endeavor, harmonizing rigorous analysis with methodological integrity, all within the pursuit of unraveling the intricate genetic underpinnings of bladder cancer within the Asian population.

## 3. Results

### 3.1. Literature Search

**Figure 1:**
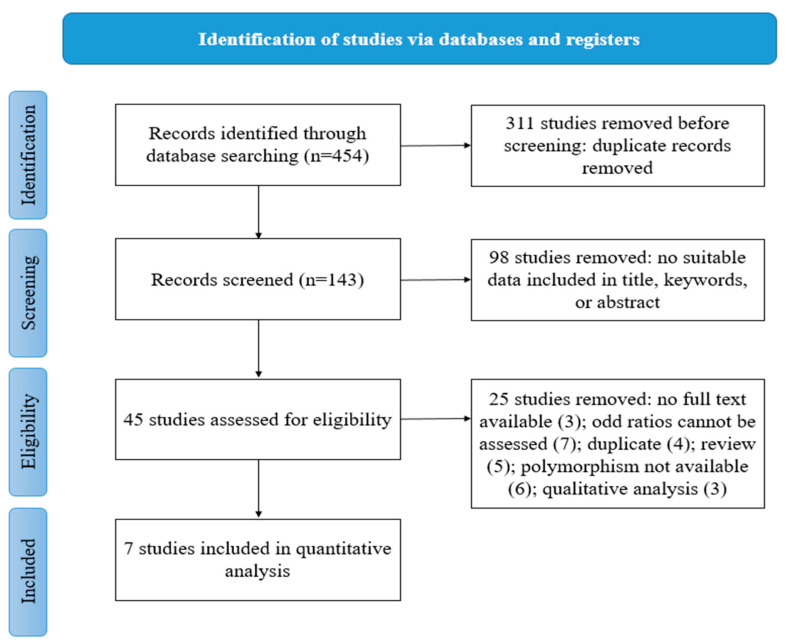
Flow chart of study selection.

The database search yielded a total of 454 records. Following the elimination of duplicate entries, a total of 143 records were subjected to analysis on the basis of their respective titles and abstracts. Ninety-eight publications were excluded from the study due to their failure to assess the polymorphism of interest, lack of relevance to bladder cancer, being classified as reviews, or having no association with the epigenetic interest of the study. Subsequent to that, a total of 45 articles in their entirety were deemed eligible based on the established criteria for eligibility, with 38 of them being subsequently excluded. Ultimately, a total of seven studies were deemed suitable for inclusion in the meta-analysis, as indicated by the references cited [16,17,18,19,20,21,22]. The selection process, as illustrated in Figure 1, was conducted in accordance with the “PRISMA 2020 flow diagram for new systematic reviews.” The search was limited to databases and registers exclusively [23].

### 3.2. Characteristics of the Included Study

Table 1 outlines the primary attributes of the studies that were incorporated in the analysis. The studies incorporated populations of Asian descent originating from various regions, including South, East, and West Asia. The aforementioned regions comprise Pakistan, China, Taiwan, India, Turkey, and Iran. All the papers incorporated in the study employed a uniform genotyping technique, namely polymerase chain reaction-restriction fragment length polymorphism. The conducted meta-analysis assessed the quality of the seven studies that were included, and the scores obtained ranged from 2 to 11. A score of 9, indicating high quality, was attained in 43% of the papers that were included. The studies incorporated in the current meta-analysis exhibited Hardy–Weinberg equilibrium and had adjusted p-values exceeding 0.05.

### 3.3. Association between Methylenetetrahydrofolate Reductase SNPs and Bladder Cancer and Accuracy Test

**Figure 2:**
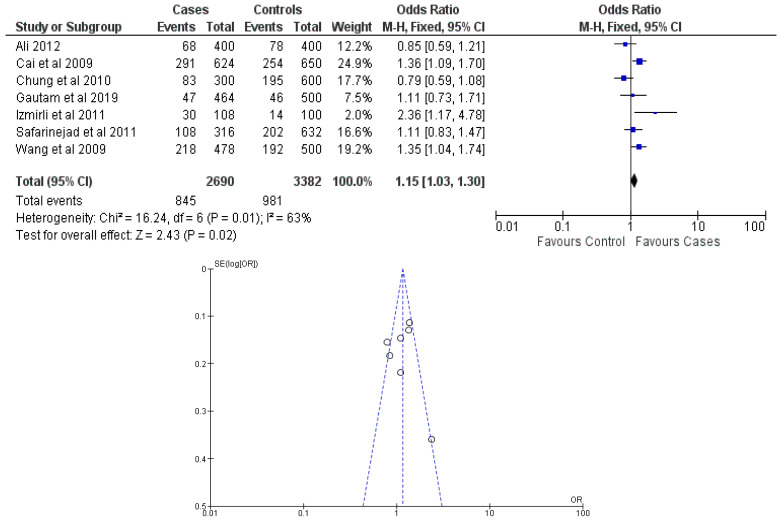
Forest plot and funnel plot of T vs. C allele analysis [16,17,18,19,20,21,22].

**Figure 3:**
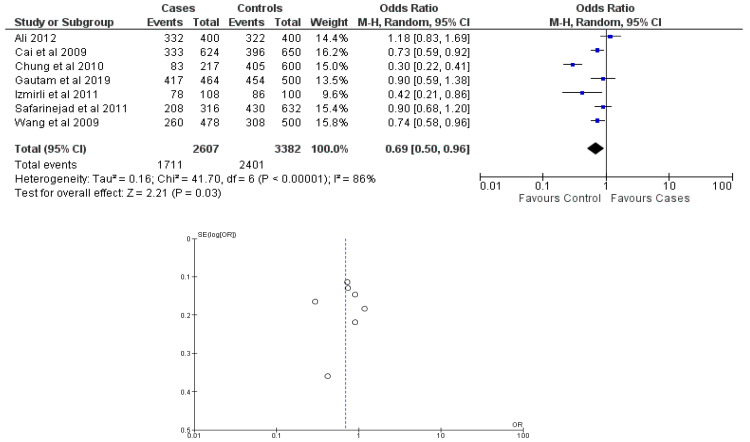
Forest plot and funnel plot of C vs. T allele analysis [16,17,18,19,20,21,22].

**Figure 4:**
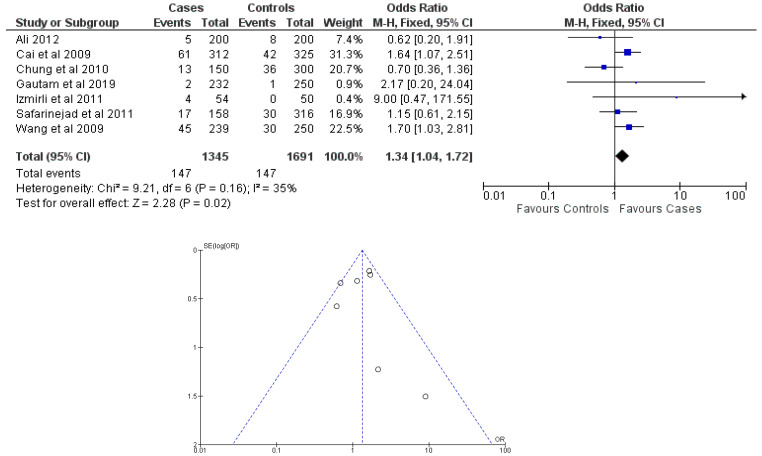
Forest plot and funnel plot of TT vs. TC + CC genotype analysis [16,17,18,19,20,21,22].

**Figure 5:**
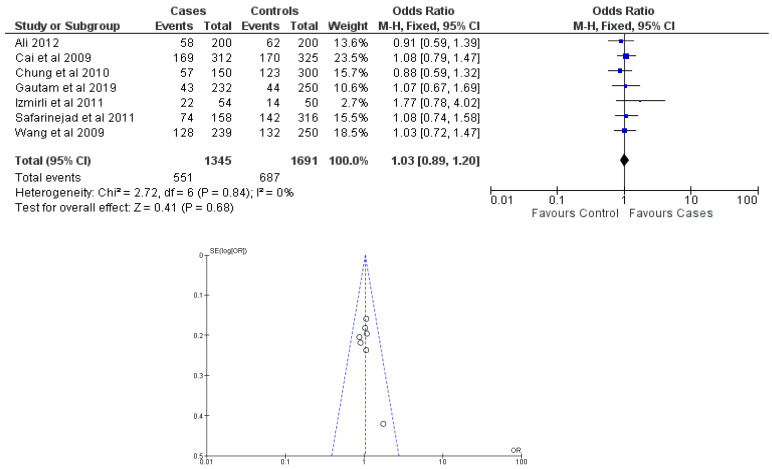
Forest plot and funnel plot of TC vs. TT + CC genotype analysis [16,17,18,19,20,21,22].

**Figure 6:**
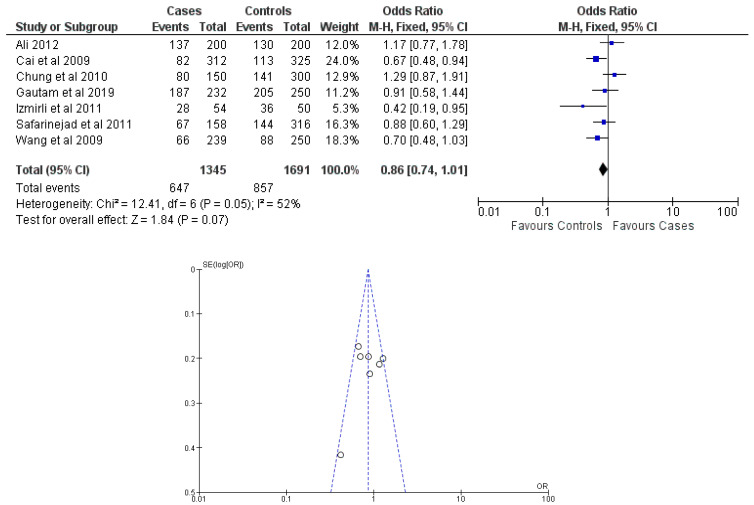
Forest plot and funnel plot of CC vs. TC + TT genotype analysis [16,17,18,19,20,21,22].

There were seven total research that made the result. There was a correlation established between polymorphisms in the gene methylenetetrahydrofolate reductase C677T (rs1801133) and an increased risk of bladder cancer. It was found that SNPs that had either the T-allele or the TT genotype were more likely to be related with the event in question. The odds ratio (OR) of the T allele over the C allele was 1.15 in the overall analysis of the Asian population (95 percent confidence interval [CI]: 1.03–1.30; *p*-value = 0.03), and the OR of the TT genotype over the TC+CC genotype was 1.34 (95 percent confidence interval [CI]: 1.04–1.72; *p*-value = 0.02). On the other hand, there was no association between the CC genotype and bladder cancer that could be found. A fascinating result that emerged from this study was that while epigenetic findings had a low sensitivity that varied from 11 to 68%, their specificity was good, reaching up to 91.31% for the detection of TT genotypes. Specifically, we employed a comprehensive dataset that encompassed a wide range of scenarios and variations. To determine sensitivity, we calculated the proportion of actual positive cases correctly identified by the method. This involved comparing the method’s predictions against a gold standard set of positive cases. For specificity, we measured the proportion of actual negative cases correctly identified as negative by the method. This evaluation was carried out by comparing the method’s predictions against a reference set of negative cases. The results are shown in Table 2. Forest plots and funnel plots reporting each analysis were reported in Figure 2, Figure 3, Figure 4, Figure 5 and Figure 6.

### 3.4. Publication Bias

The study conducted an evaluation of publication bias for each genetic model through the utilization of a funnel plot, as well as an example of allele contrast and Egger’s test, as presented in Table 3, respectively. The present meta-analysis findings indicate that no publication bias was detected among the studies included for the methylenetetrahydrofolate reductase SNP rs1801133, as evidenced by the Egger’s test and funnel plots for the SNP.

## 4. Discussion

The study of single-nucleotide polymorphisms (SNPs) emerges as a notably promising avenue within the ambit of precision medicine, a trajectory that holds immense potential both for immunological disorders and oncological causation [24]. However, the application of epigenetic analysis, coupled with the subsequent pursuit of meta-analysis, is still navigating the contours of its popularity. While systematic reviews predominantly find their foothold in clinical setting comparisons and evaluations of natural compound studies [25,26,27,28,29,30], our research undertook a meticulously orchestrated systematic review, aimed at penetrating the intricate realm of the association between the methylenetetrahydrofolate reductase rs1801133 polymorphism and the incidence of bladder cancer (BC).

The discoveries stemming from the meta-analysis cast a spotlight on noteworthy correlations with rs1801133 within the Asian demographic, particularly the comparison between TT and TC + CC genotypes. Notably, the genotyping method PCR-RFLP emerged as a commonly harnessed technique that has succeeded in tracing a discernible correlation between bladder cancer and rs1801133 [T vs. C; TT vs. TC + CC].

The comprehensive analysis undertaken not only accentuates a significant correlation between the rs1801133 polymorphism and the predisposition to BC, but also echoes findings from antecedent research endeavors. These previous studies have notably identified this polymorphism as a potential risk factor for other malignancies, including lung cancer [31] and esophageal cancer [32]. However, it is worth mentioning that the pursuit of correlation between the rs1801133 polymorphism of methylenetetrahydrofolate reductase and breast cancer susceptibility has produced varied outcomes, underscoring the contextual complexities inherent in such analyses.

Within the intricate landscape of bladder cancer genetics, the phenomenon of G:C to A:T transitions emerges as a key point of interest, with reference to the inherent deamination of cytosine and 5-methylcytosine residues. These occurrences underscore the intrinsic complexity of genetic variations influencing cancer predisposition. These types of alterations account for around 37% of the mutations affecting the TP53 tumour suppressor gene in breast cancer, as reported in reference [33]. Several studies have indicated that roughly 50% of samples of MIBC (muscle invasive bladder cancer) display a mutation in TP53, with 76% of the samples exhibiting functional inactivation, as reported in reference [34].

Drawing from established research narratives, the equilibrium of 5,10-CH2-THF utilization, essential for methionine and purine synthesis, is closely entwined with the thermolabile 677 T variant. As per the findings reported in reference [35], the enzyme activity levels of individuals possessing heterozygous and homozygous mutations are 67% and 25%, respectively, in comparison to those harbouring the wild-type CC. The T allele has been observed to prioritise DNA synthesis over the methionine cycle in the presence of adequate folate levels.

Delving further, it has been discerned that the rs1801133 variant might exert an impact on CpG methylation by limiting the availability of methyl groups, consequently culminating in DNA hypomethylation [36]. This profound occurrence bears implications for DNA stability and introduces a novel perspective on the correlation between methylenetetrahydrofolate reductase variants and cancer susceptibility [37].

The gravitas of our study resonates within the broader spectrum of bladder cancer research, aligning itself with the aspiration of comprehensively unraveling the molecular intricacies underpinning this ailment. This pursuit extends beyond mere academic curiosity, seamlessly aligning with the aspiration of identifying therapeutic targets and fostering precision medicine strategies [38]. Furthermore, our work draws inspiration from demographic considerations, resonating with insights akin to those gleaned from Ferro et al.’s study [39].

In light of these research endeavors, our study enriches the understanding of genetic variations within the context of bladder cancer susceptibility in the Asian population. This landmark research marks the inaugural foray into meta-analysis concerning BC susceptibility within the Asian demographic, magnifying its significance. Nonetheless, it is crucial to acknowledge the constraints of our investigation, particularly the limitation imposed by the language barrier that might have inadvertently excluded valuable contributions published in languages other than English.

## 5. Conclusions

Methylenetetrahydrofolate reductase T-allele/TT genotype has been linked to an increased risk of bladder cancer in Asian populations. More research in a wider variety of populations is needed before it can be established whether or not the rs1801133 SNP used for this study has an influence on the incidence of BC dependent on ethnicity or location. In order to confirm whether or not SNPs as rs1801133 may have a role in the development of bladder cancer, more research has to be conducted in countries where it is not sufficiently represented, including in Asian continent.

## Figures and Tables

**Table 1:** Characteristics of the 7 studies included in the methylenetetrahydrofolate reductase C677T (rs1801133) polymorphisms epigenetic meta-analysis.

Author & Year	Country	SNP	Genotype	Case	Control	Total Samples	Quality Score
Ali, 2012 [22]	Pakistan	rs1801133	PCR-RFLP	200	200	400	10
Cai et al., 2009 [17]	China	rs1801133	PCR-RFLP	312	325	637	7
Chung et al., 2010 [19]	Taiwan	rs1801133	PCR-RFLP	150	300	450	8
Gautam et al., 2019 [20]	India	rs1801133	PCR-RFLP	232	250	482	7
Izmirli et al., 2011 [16]	Turkey	rs1801133	PCR-RFLP	54	50	104	7
Safarinejad et al., 2011 [18]	Iran	rs1801133	PCR-RFLP	158	316	474	9
Wang et al., 2009 [21]	China	rs1801133	PCR-RFLP	239	250	489	9

**Table 2:** Summary of case and control genotype results.

Author & Year	Case							Control							HWE Adjusted Value
	TT	TC	CC	N	T	C	n	TT	TC	CC	N	T	C	n	
Ali, 2012 [22]	5	58	137	200	68	332	400	8	62	130	200	78	8	400	0.9125
Cai et al., 2009 [17]	61	169	82	312	291	333	624	42	170	113	325	254	396	650	0.3808
Chung et al., 2010 [19]	13	57	80	150	83	217	300	36	123	141	300	195	405	600	0.6226
Gautam et al., 2019 [20]	2	43	187	232	47	417	464	1	44	205	250	46	454	500	0.7084
Izmirli et al., 2011 [16]	4	22	28	54	30	78	108	0	14	36	50	14	86	100	0.6226
Safarinejad et al., 2011 [18]	17	74	67	158	108	208	316	30	142	144	316	202	430	632	0.7258
Wang et al., 2009 [21]	45	128	66	239	218	260	478	30	132	88	250	192	308	500	0.3808

**Table 3:** Summary of the association methylenetetrahydrofolate reductase C677T (rs1801133) polymorphisms and increased risk of bladder cancer.

Allele & Genotype	NS	Model	Sensitivity, %	Specificity, %	OR	95%CI	Chi^2^	I^2^	*p*-Value	Egger’s Test *p*-Value
rs1801133 C677T										
C vs. T	7	Fixed	68.59	29.01	0.69	0.50–0.96	16.24	60%	0.02 *	0.370
T vs. C	7	Random	31.41	68.59	1.15	1.03–1.30	41.70	86%	0.03 *	0.630
TT vs. TC + CC	7	Fixed	10.93	91.31	1.34	1.04–1.72	9.21	35%	0.02 *	0.916
TC vs. TT + CC	7	Fixed	40.97	59.37	1.03	0.89–1.20	2.72	0%	0.68	0.810
CC vs. TC + TT	7	Fixed	48.10	49.32	0.86	0.74–1.01	12.41	52%	0.07	0.397

*, *p* < 0.05. NS: number of studies; OR: odds ratio; CI: confidence interval; pH: p-heterogeneity; I^2^: heterogeneity; *p*: overall analysis *p*-value.

## Data Availability

Publicly available studies were used in this systematic review.
